# The Relevance of Foreshocks in Earthquake Triggering: A Statistical Study

**DOI:** 10.3390/e21020173

**Published:** 2019-02-13

**Authors:** Eugenio Lippiello, Cataldo Godano, Lucilla de Arcangelis

**Affiliations:** 1 Department of Mathematics and Physics, University of Campania “L. Vanvitelli”, Viale Lincoln 5, 81100 Caserta, Italy; 2 Department of Engineering, University of Campania “L. Vanvitelli’, Via Roma 29, 81031 Aversa (CE), Italy

**Keywords:** forecasting, foreshocks, ETAS model

## Abstract

An increase of seismic activity is often observed before large earthquakes. Events responsible for this increase are usually named foreshock and their occurrence probably represents the most reliable precursory pattern. Many foreshocks statistical features can be interpreted in terms of the standard mainshock-to-aftershock triggering process and are recovered in the Epidemic Type Aftershock Sequence ETAS model. Here we present a statistical study of instrumental seismic catalogs from four different geographic regions. We focus on some common features of foreshocks in the four catalogs which cannot be reproduced by the ETAS model. In particular we find in instrumental catalogs a significantly larger number of foreshocks than the one predicted by the ETAS model. We show that this foreshock excess cannot be attributed to catalog incompleteness. We therefore propose a generalized formulation of the ETAS model, the ETAFS model, which explicitly includes foreshock occurrence. Statistical features of aftershocks and foreshocks in the ETAFS model are in very good agreement with instrumental results.

## 1. Introduction

The epidemic-type-aftershock sequence ETAS model [1,2,3] is presently considered “a de facto standard model, or null hypotheses, for other models and ideas to be compared to” [4]. The model assumes that two classes of earthquakes exist: Independent background and triggered earthquakes. An epidemic organization of events arises under the assumption that each earthquake can trigger its own descendants leading to a branching organization. From a physical point of view, background seismicity can be thought as the effect of the slow tectonic drive whereas triggered earthquakes are induced by stress redistribution after previous shocks. The interplay between background and triggered seismicity leads to non trivial spatio-temporal patterns which can be highlighted by the complex behavior of the inter-event time distribution [5,6,7,8,9] and are also evident in natural time analysis [10,11] (see ref. [12] for a Review). In the ETAS model the occurrence rate of triggered events is obtained on the basis of well established empirical laws controlling the spatio-temporal clustering of aftershocks. More precisely:

- A1: The number of aftershocks $n_{a}$ depends on the mainshock magnitude $m_{M}$ according to the productivity law $n_{a}=K_{a}10^{\alpha_{a}m_{M}}$;
- A2: The aftershock number decays as function of the time Δt from the mainshock, consistently with the Omori law $n_{a}\left(\Delta t\right)∼\Delta t^{-p}$ with p≃1;
- A3: The distribution of epicentral distances between mainshock and aftershocks $G(\Delta r,m_{M})$ clearly depends on the mainshock magnitude $m_{M}$.

These laws are implemented in a branching process where each event can trigger its own aftershocks. By construction, the ETAS model is very efficient in reproducing statistical features (A1–A3) of aftershock organization, observed in instrumental catalogs. At the same time, in the ETAS model an event can trigger also a larger shock. In this situation the triggering event is often named “foreshock” and the triggered earthquake, if it is the largest event in the sequence, is named “mainshock”.

In this study, we adopt the standard definition of mainshocks as events sufficiently isolated in time and space from other larger events. Foreshocks (aftershocks) are then all events occurring close in space and in time before (after) the mainshock. We wish to stress that within the ETAS framework, this classification of events does not reflect different physical properties since, as anticipated, only two kinds (independent or triggered) earthquakes are assumed and, for instance, a mainshock can be either an independent or a triggered earthquake. On the other hand, according to a nucleation theory [13,14,15], the nucleation phase can be characterized by the occurrence of smaller earthquakes inside the region involved in the fracture process of the subsequent incoming larger shock. This pre-shock seismicity is not implemented in the ETAS model and the main question addressed in this study is if its inclusion, within the ETAS modeling, gives a more accurate description of foreshock organization in instrumental catalogs.

In recent years, different studies have enlightened some differences between statistical features of foreshocks in instrumental and in ETAS catalogs [12,16,17,18,19,20,21,22,23,24,25,26,27,28,29,30]. In this study we will focus on the two main differences:

- F1: The average foreshock number in instrumental catalogs is significantly larger than the one expected according to the ETAS model;
- F2: The organization in space of instrumental foreshocks exhibit a dependence on the mainshock magnitude not predicted by the ETAS model.

In our study, we first investigate whether the incompleteness of instrumental data sets can justify the above differences. We will show that none of the two features can be attributed to spurious incompleteness. This motivates a generalization of the ETAS model, the ETAFS model, which implements the hypothesis that each event can be anticipated by a cluster of smaller earthquakes. This extra ingredient allows us to generate numerical ETAFS catalogs with statistical features of foreshocks in agreement with instrumental catalogs. In the following section we recall the ETAS model and introduce its generalizations considered in this study. Section 3 presents statistical features of foreshocks and aftershocks in instrumental catalogs. In the subsequent section these features are compared with those obtained in numerical catalogs. Conclusions are drawn in the final section.

## 2. Epidemic Models for Aftershocks and Foreshock Occurrence

### 2.1. The ETAS Model

The ETAS model is specified by the conditional intensity function, which represents the expected seismicity rate in a given space position conditioned to a given observational history. The conditional intensity function $\Lambda (m,\vec{x},t)$, which represents the occurrence probability of events with magnitude $m\geq m_{c}$ in the position $\vec{x}$ at time *t*, can be written in the following form:

(1) $$\Lambda \left(m,\vec{r},t\right)=\left[\mu w\left(\vec{r}\right)+\sum_{i:t_{i}<t}\left(|{\vec{r}}_{i}-\vec{r}|,t-t_{i},m_{i}\right)\right]\frac{1}{b\log(10)}10^{-b(m-m_{c})},$$

where $\mu w(\vec{r})$ is a time independent contribution which reflects the spatial distribution of background seismicity. In the ETAS model one assumes that aftershock occurrence time, epicentral coordinates and magnitude are independent variables and the form of the spatio-temporal kernel $Q(\Delta r_{i},t-t_{i},m_{i})$ is obtained implementing the three well established laws for aftershock triggering (A1–A3) leading to

(2) $$\Lambda_{ETAS}\left(m,\vec{r},t\right)=\left[\mu w\left(\vec{r}\right)+\frac{A(p-1)}{c}\sum_{i:t_{i}<t}10^{\alpha (m_{i}-m_{c})}{\left(1+\frac{t-t_{i}}{c}\right)}^{-p}G\left(\Delta r_{i},m_{i}\right)\right]\frac{1}{b\log(10)}10^{-b(m-m_{c})},$$

where $\Delta r_{i}=\left|{\vec{r}}_{i}-\vec{r}\right|$ and the sum extends over all events with magnitude $m_{i}$, epicentral coordinates ${\vec{x}}_{i}$ and occurrence time $t_{i}<t$. Here, $G(\Delta r_{i},m_{i})$ is the spatial distribution of aftershock epicentral distances in instrumental catalogs obtained from feature A3.

The first step in the numerical simulation is the generation of the background independent events. To this extent, we initially fix the temporal duration of the catalog *T* so that $N_{m}=\mu T$ independent events are randomly located within the temporal interval [0,T]. For the spatial position of the background events, we construct a fine space-covering grid of $N_{c}$ cells and events are located within a cell with probability $w({\vec{r}}_{i})$ where ${\vec{r}}_{i}$ is the position of the cell center. We assign a magnitude $m\geq m_{c}$ to each event according to the Gutenberg-Richter law $p\left(m\right)=\frac{1}{b\log(10)}10^{-b(m-m_{c})}$. The second step is the “first generation” of aftershocks that we obtain associating to each event *i*, generated at the previous step, several aftershocks $n_{a}\left(m_{i}\right)$. We extract the value of $n_{a}\left(m_{i}\right)$ from a Poisson distribution with average $A10^{\alpha (m_{i}-m_{c})}$. The temporal and spatial distance from its mother event of a first-order generation aftershocks is randomly extracted according to features A2 and A3, respectively. More precisely, we obtain occurrence time $\Delta t=t-t_{i}$ from the Omori-Utsu law (A2) and the epicentral distance $\Delta r_{i}$ according to the procedure described in Lippiello et al. [29]. This procedure allows us to implement in numerical simulations the spatial distribution of aftershocks measured in the instrumental catalog. We also assume an isotropic aftershock distribution whereas magnitudes are always assigned according to the Gutenberg-Richter law. Once all first generation aftershocks have been triggered, we iterate the process at the subsequent step in order to determine the second order generation of aftershocks considering as mother event the first order aftershocks. We then iterate the process until no further aftershocks are triggered. A final sorting procedure is applied to reorder all events according to their occurrence time.

### 2.2. The ETAS Incomplete Catalog

Because of the overlap of seismic coda waves, many aftershocks are not recorded in particular in the first temporal periods after large shocks [31,32,33,34,35,36,37,38,39,40]. The direct inspection of seismic signals [37,40] has shown that at a temporal distance τ after an event of magnitude $m_{0}$, there exists a lower magnitude level $m_{x}\left(\tau ,m_{0}\right)$ such that it is impossible to detect events with $m\leq m_{x}\left(\tau ,m_{0}\right)$. Results indicate a logarithmic decay of $m_{x}\left(\tau ,m_{0}\right)$ in time

(3) $$m_{x}\left(\tau ,m_{0}\right)=m_{0}-\psi \log\left(\tau \right)-\Delta m$$

with ψ≃1 and Δm≃1, if τ is measured in seconds. Accordingly, earthquakes can be hidden by larger events preceding them at small temporal distances.

In our study we take explicitly into account the aftershock incompleteness adopting the same procedure developed in [40] to reproduce both the non-trivial dependence of the *c*-value in the OU law on the mainshock magnitude [8,41,42,43,44,45,46], as well as the non trivial magnitude correlations between subsequent earthquakes [11,47,48,49,50,51,52,53]. The model, defined as ETASI2 model, implements aftershock incompleteness by multiplying the occurrence rate $Q(\Delta r,t-t_{i},m_{i})$ in Equation (1) by a detection rate function of the magnitude Φ(m|q,σ) represented by the cumulative distribution function of the normal distribution. The function Φ(m|q,σ) depends on two parameters *q* and σ representing, respectively, the magnitude with a 50% detection rate and a partially detected magnitude range. In other words, Φ(q|q,σ)=0.5 whereas Φ(m|q,σ)≃0 when m<q−σ and Φ(m|q,σ)≃1 when m>q−σ. In the ETASI2 model the *q* parameter depends on time according to Equation (3), $q=m_{x}\left(\tau ,m_{i}\right)$.

Numerical ETASI2 catalogs are obtained starting from the complete data set generated via the standard ETAS model. For each couple of events in the ETAS catalog, with magnitudes $m_{i},m_{j}$ and occurrence times $t_{i}>t_{j}$, we evaluate the quantity $q_{ij}=m_{x}\left(t_{i}-t_{j},m_{j}\right)$ with $m_{x}\left(\tau ,m_{0}\right)$ given in Equation (3). The event $t_{i},m_{i}$ is then removed from the catalog with a probability $\prod_{j}\left[1-\Phi (m_{i}|q_{ij},\sigma )\right]$ where the product extends over all events with $t_{j}<t_{i}$ occurred within a radius of 100 km from the epicenter of the *i*-th earthquake.

### 2.3. The ETAFS Model

In this study, we propose a novel model, the Epidemic Type Aftershocks and Foreshock Sequence (ETAFS) model. The model, together with the standard aftershock triggering, assumes that events can be also anticipated by a cluster of smaller events not assumed in the ETAS model. More precisely, in the ETAFS model aftershocks are triggered with the same occurrence probability $\Lambda_{ETAS}$ of the ETAS model (Equation (2)). The new ingredient is that each earthquake can be also anticipated by several foreshocks leading to

(4) $$\Lambda_{ETAFS}\left(m,\vec{r},t\right)=\Lambda_{ETAS}\left(m,\vec{r},t\right)+\sum_{i:t_{i}>t}^{ETAS}Q_{f}\left(|{\vec{r}}_{i}-\vec{r}|,t-t_{i},m_{i}\right)\frac{1}{b\log(10)}10^{-b(m-m_{c})},$$

with

(5) $$Q_{f}\left(\Delta r,t-t_{i},m_{i}\right)=\frac{B(p-1)}{c^{\prime }}10^{\alpha^{\prime }\left(m_{i}-m_{c}\right)}{\left(1+\frac{t_{i}-t}{c^{\prime }}\right)}^{-p}G_{f}\left(\Delta r_{i},m_{i}\right),$$

where the sum is restricted to events occurred at time $t_{i}>t$ according to the first addend in Equation (4) $\Lambda_{ETAS}$. We assume that the spatial organization of foreshocks is similar to the aftershock one and then we take $G_{f}\left(\Delta r_{i},m_{i}\right)=G\left(\Delta r_{i},m_{i}\right)$ in Equation (2). Moreover we implement an inverse-Omori law with the same *p* as for aftershock occurrence, to reduce the number of model parameters. This choice has no physical justification for it and we expect that similar results can be recovered with other functional forms of temporal clustering.

The organization in time and space of events triggered according to kernel $Q_{f}$ in Equation (4), as well as their number, depends on the occurrence time, location and magnitude of the incoming larger event. This violates time causality since the occurrence probability of an event depends on future events. However, from the point of view of point-processes, the model is well defined and the second addend $Q_{f}$ in Equation (4) can be simply viewed as a further generation step in the branching process. More precisely. The ETAFS catalog is simulated completing all the aftershock generation steps of the ETAS model, according exactly to the procedure outlined in Section 2.1, and assuming a further generation step which correspond to the generation of foreshocks. These are generated with the same rules implemented for aftershocks with the difference that the sign of $\Delta t=t-t_{i}$ must be subsequently inverted. Magnitudes are again extracted from the Gutenberg-Richter law but with the additional constraint that the foreshock magnitude must be smaller than $m_{i}$. We stress that the process of foreshock generation is not iterated: A foreshock does not trigger other aftershocks and is not anticipated by other foreshocks.

Catalog incompleteness can be also taken into account within the ETAFS model by applying the same procedure outlined in Section 2.2 to obtain ETASI2 from ETAS catalogs.

## 3. Results in the Instrumental Catalogs

### 3.1. Data Sets and the Definitions of Mainshocks, Aftershocks and Foreshocks

We perform a systematic analysis of four different instrumental catalogs: The relocated Southern California earthquake catalog (RSCEC) [54] (from 01/01/1981 to 12/31/2013), the relocated Northern California earthquake catalog (RNCEC) [55] (from 01/01/1981 to 06/30/2011), the Italian earthquake catalog (ItEC) [56] (from 01/01/2002 to 12/31/2012) and the Japanese earthquake catalog (JaEC) [57] (from 01/01/1966 to 01/30/2011). We use the same definition of mainshock, aftershocks and foreshocks adopted in [29]. More precisely, we define an event as “mainshock” if a larger earthquake does not occur in the previous *y* days and within a distance *L*. In addition a larger earthquake must not occur in the selected area in the following $y_{2}$ days. We then associate to each mainshock its own “aftershocks” and “foreshocks” defined as all earthquakes recorded in the subsequent or in the preceding time interval T=12 h, respectively, and within a circle of radius $R\leq R_{M}$ centered in the mainshock epicenter. We use different $R_{M}$ for different catalogs: $R_{M}=2$ km for RSCEC and RNEC, $R_{M}=5$ km for ItEC and $R_{M}=10$ km for JaEC.

The choice of parameters has been deeply investigated in previous studies [17,29,50,58] and here we implement typical values, L=100 km, y=3 and $y_{2}=0.5$. The value of $R_{M}$ is fixed imposing that for each instrumental catalog, different choices of T≤12 h produces similar results $\rho_{a}\left(\Delta r,m_{M}\right)$ when $\Delta r<R_{M}$. This leads to $R_{M}=2,2,5,10$ km, for RSCEC, NSCEC; ItEC and JMAC, respectively (see Figure 17 in [29]). We observe that the temporal interval considered for aftershock and foreshock occurrence [−T,T] is always included in the temporal interval $\left[-y,y_{2}\right]$ days, where events larger than the mainshock cannot occur. This choice therefore implies that aftershocks and foreshocks are assumed to be smaller than the mainshock, by definition.

Once mainshocks are identified, they are grouped in classes according to their magnitude $m\in [m_{M},m_{M}+1)$ and, for each catalog, we evaluate the total number of mainshocks belonging to the given class $n_{main}\left(m_{M}\right)$, the total number of aftershocks $n_{aft}\left(m_{M}\right)$ which follow mainshocks in the given class and the total number of foreshocks $n_{fore}\left(m_{M}\right)$ which anticipate mainshocks in the given class. We also evaluate the epicentral distance Δr between each main-aftershock and main-foreshock couple and construct the aftershock and foreshock epicentral distance distributions, $\rho_{a}\left(\Delta r,m_{M}\right)$ and $\rho_{f}\left(\Delta r,m_{M}\right)$.

### 3.2. The Aftershock and Foreshock Number

In our study, we consider all events with magnitude above a magnitude threshold $m_{th}=2$. The lower threshold $m_{th}$ must not be confused with $m_{c}$ in Equation (2). Indeed, $m_{c}$ is a fixed parameter of the ETAS model and synthetic catalogs contain only events with $m\geq m_{c}$. The lower magnitude $m_{th}$, conversely, is a parameter implemented in the data analysis and it can be arbitrarily varied with $m_{th}\geq m_{c}$.

**Figure 1:**
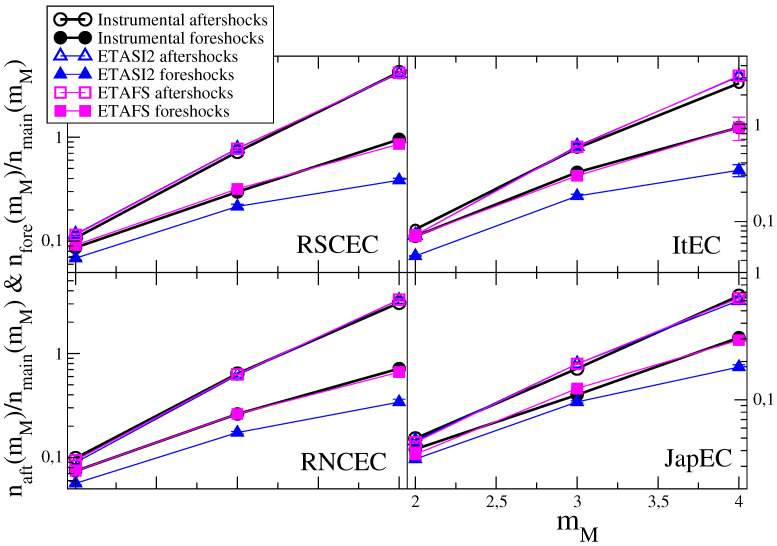
(Color online) The ratio $n_{aft}\left(m_{M}\right)/n_{main}\left(m_{M}\right)$ and $n_{fore}\left(m_{M}\right)/n_{main}\left(m_{M}\right)$ in instrumental and synthetic catalogs. Different panels correspond to different instrumental catalogs. We use open symbols for $n_{aft}\left(m_{M}\right)/n_{main}\left(m_{M}\right)$ and filled symbols for $n_{fore}\left(m_{M}\right)/n_{main}\left(m_{M}\right)$. Results from the instrumental data sets are indicated with black circles. Green triangles are results for the ETASI2 model and red squares for the ETAFS model. The error bars (of the same size of symbols) in numerical catalogs represent the standard deviation from 100 realization of synthetic catalogs. The best parameter of the ETAFS model are listed in Table 1 whereas for the ETASI2 model the best agreement is obtained with A=0.084, α=0.9 and $\mu =5.85\times 10^{-4}\;s^{-1}$ for RSCEC, A=0.082, α=0.88 and $\mu =4.98\times 10^{-4}\;s^{-1}$ for RNCEC, A=0.082, α=0.88 and $\mu =5.21\times 10^{-4}\;s^{-1}$ for ItEC and A=0.26, α=0.60 and $\mu =5.92\times 10^{-3}\;s^{-1}$ for JapEC.

In Figure 1, we plot the ratio between aftershock and mainshock number $n_{aft}\left(m_{M}\right)/n_{main}\left(m_{M}\right)$ for different mainshock classes $m_{M}$ and for the different instrumental catalogs. We also plot the ratio between foreshock and mainshock number $n_{fore}\left(m_{M}\right)/n_{main}\left(m_{M}\right)$.

Results in Figure 1 show that the aftershock number is systematically larger than the foreshock number and this difference increases for increasing $m_{M}$. The aftershock number is consistent with the Utsu-productivity law (A1)

(6) $$n_{aft}\left(m_{M}\right)/n_{main}\left(m_{M}\right)=K_{a}10^{\alpha m_{M}}$$

and a similar law is also observed for foreshocks $n_{fore}\left(m_{M}\right)/n_{main}\left(m_{M}\right)=K_{f}10^{\alpha_{f}m_{M}}$.

### 3.3. Aftershock and Foreshock Spatial Distribution

**Figure 2:**
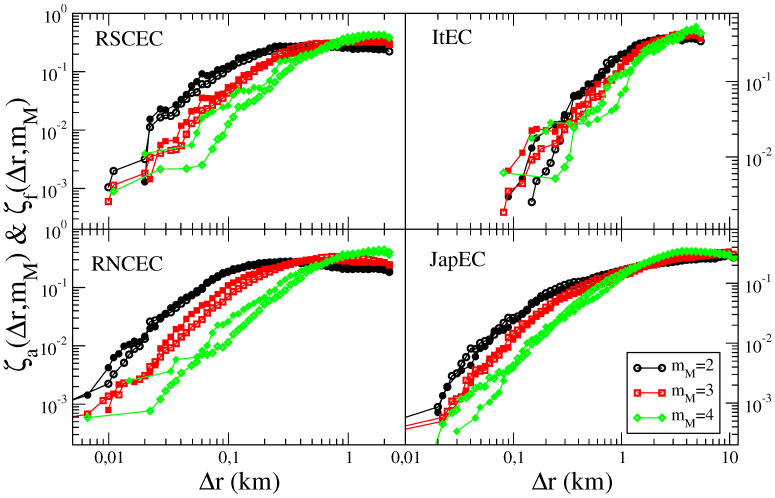
(Color online) The average distance $\zeta_{a}\left(\Delta r,m_{M}\right)$ of aftershocks (open symbols) and of foreshocks $\zeta_{f}\left(\Delta r,m_{M}\right)$ (filled symbols) is plotted as function of Δr for the different catalogs. Different mainshock magnitude classes are plotted with different colors and symbols.

In Figure 2 we plot, for different catalogs, the average epicentral distance $\zeta_{a}\left(\Delta r,m_{M}\right)$ defined as as $\zeta_{a}\left(\Delta r,m_{M}\right)\equiv \frac{1}{\Delta r}\int_{0}^{\Delta r}d\left(\Delta r^{\prime }\right)\Delta r^{\prime }\rho_{a}\left(\Delta r^{\prime },m_{M}\right)$, were $\rho_{a}\left(\Delta r,m_{M}\right)$ is the aftershock epicentral distribution. We also define the average foreshock epicentral distance

$$\zeta_{f}\left(\Delta r,m_{M}\right)\equiv \frac{1}{\Delta r}\int_{0}^{\Delta r}d\left(\Delta r^{\prime }\right)\Delta r\rho_{f}\left(\Delta r^{\prime },m_{M}\right)$$

where $\rho_{f}\left(\Delta r,m_{M}\right)$ is the foreshock epicentral distribution.

Figure 2 shows that for all catalogs, data corresponding to different $m_{M}$ are well separated and in all cases $\zeta_{a}\left(\Delta r,m_{M}\right)≃\zeta_{f}\left(\Delta r,m_{M}\right)$. The latter result is a direct consequence of the similarity $\rho_{a}\left(\Delta r,m_{M}\right)≃\rho_{f}\left(\Delta r,m_{M}\right)$ [29].

## 4. Results in Numerical Catalogs

### 4.1. Results in the ETAS Catalog

#### The Aftershock and Foreshock Number in the ETAS Catalog

In Figure 1, we compare results for $n_{aft}\left(m_{M}\right)/n_{main}\left(m_{M}\right)$ and $n_{fore}\left(m_{M}\right)/n_{main}\left(m_{M}\right)$ with the results obtained applying the same definition of aftershocks, mainshocks and foreshocks to ETAS catalogs. The values of both $n_{aft}\left(m_{M}\right)/n_{main}\left(m_{M}\right)$ and $n_{fore}\left(m_{M}\right)/n_{main}\left(m_{M}\right)$ depend on the parameters A,p,c,α (Equation (2)) of the numerical model. In particular, in first approximation, neglecting the contribution μ of background events from Equation (2) we obtain

(7) $$n_{aft}\left(m_{M}\right)\propto 10^{\alpha (m_{M}-m_{c})}$$

which indicates that the ETAS model can reproduce the experimental result Equation (6) with $\alpha_{f}=\alpha$.

In numerical simulations we have explored a wide range of ETAS parameters A,p,c,α and verified that there exists a set of parameters leading to ETAS catalogs with the same behavior of $n_{aft}\left(m_{M}\right)/n_{main}\left(m_{M}\right)$ of the instrumental ones. In all cases the agreement between ETAS and instrumental catalogs is always recovered for values of $\alpha ≳\alpha_{f}$. We wish to stress the difference between α and $\alpha_{f}$: α is the model parameter which controls the productivity law in numerical simulations whereas $\alpha_{f}$ is the value obtained applying our definition of mainshock and aftershock to ETAS catalogs and then performing a fit according to Equation (6). The small discrepancies between α and $\alpha_{f}$ can be attributed to the background contribution which weakly affects data at small $m_{M}$ whereas it can be neglected for increasing $m_{M}$.

**Figure 3:**
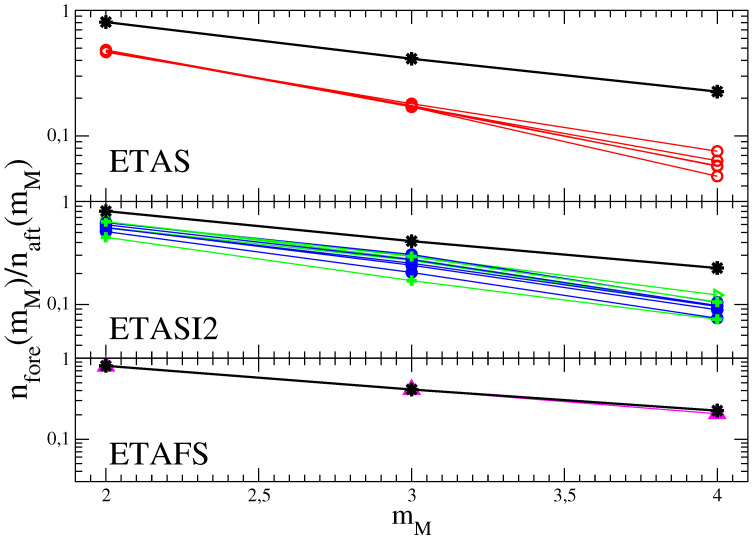
(Color online) The ratio $n_{fore}\left(m_{M}\right)/n_{aft}\left(m_{M}\right)$ in the RSCEC catalog (black stars) is compared with the value obtained in synthetic ETAS (upper panel), ETASI2 (central panel) and ETAFS (lower panel) catalog. (Upper panel) The red open symbols are results for the ETAS model for different choices of the parameters A∈[0.05,0.12], p∈[1.1,1.25] and c∈[0.001,0.1]. (Central Panel) The blue filled symbols are results for the ETASI2 model implementing Equation (3) with ϕ=0.75 and Δm=0.8 and for different choices of the parameters A∈[0.05,0.12], p∈[1.1,1.25] and c∈[0.001,0.1]. Green symbols correspond to A=0.084, c=0.01 and p=1.2 used in Figure 1 for the RSCEC catalog and considering different values of ϕ, Δm and σ. (Lower panel) The filled magenta up triangles are results of the ETAFS model with the best set of parameters listed in Table 1.

A central observation is that all choices of parameters producing agreement in $n_{aft}\left(m_{M}\right)/n_{main}\left(m_{M}\right)$ between ETAS and instrumental catalogs give a value of $n_{fore}\left(m_{M}\right)/n_{main}\left(m_{M}\right)$ in ETAS catalogs systematically smaller than the instrumental value. It is difficult to obtain a simple approximated expression for the foreshock number as function of $m_{M}$ as in Equation (7). However, it is reasonable to expect that the ratio $n_{aft}\left(m_{M}\right)/n_{fore}\left(m_{M}\right)$ is mainly controlled by the parameter α and weakly depends on the other model parameters. This is supported by numerical simulations where we fix α in order to have the same value of $\alpha_{f}$ in ETAS catalogs. Results plotted in Figure 3 show that different choices of A,p,c lead to similar results for $n_{fore}\left(m_{M}\right)/n_{aft}\left(m_{M}\right)$, in all cases, significantly smaller than the experimental value for any $m_{M}$.

### 4.2. Aftershock and Foreshock Spatial Distribution in the ETAS Catalog

**Figure 4:**
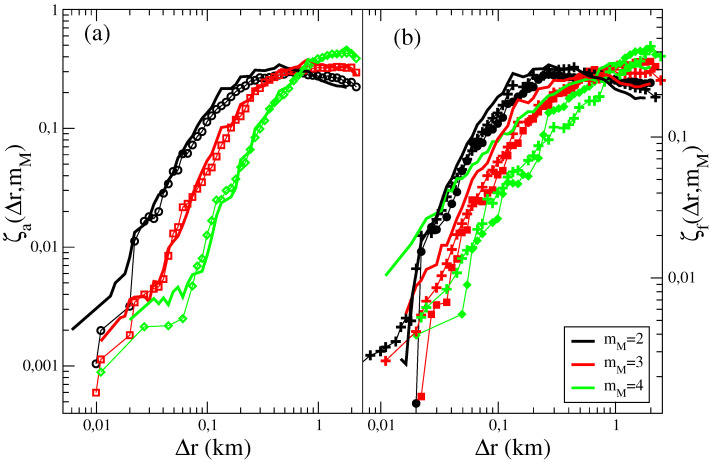
(Color online) (Left panel) The average distance of aftershocks $\zeta_{a}\left(\Delta r,m_{M}\right)$ in the RSCEC (open symbols) and in the synthetic ETASI2 catalogs (continuous lines) is plotted as function of Δr for different mainshock magnitude classes. (Right Panel) The average distance of foreshocks $\zeta_{f}\left(\Delta r,m_{M}\right)$ in the RSCEC (filled symbols) and in the synthetic ETASI2 catalog (continuous lines) is plotted as function of Δr for different mainshock magnitude classes. Results for the EATFS model, for the best set of model parameters listed in Table 1, are plotted with crosses.

The average spatial distribution of aftershocks and foreshocks in ETAS catalogs is plotted in Figure 4. Results show that even if one can generate ETAS catalogs with $\zeta_{a}\left(\Delta r,m_{M}\right)$ in good agreement with instrumental catalogs (Figure 4a), significant differences are observed between the numeric and the experimental $\zeta_{f}\left(\Delta r,m_{M}\right)$ (Figure 4b). This difference becomes more pronounced for increasing $m_{M}$ and can simply attributed to the nature of foreshocks in the ETAS model which are typical events that have triggered a larger shock. Indeed, neglecting the contribution of background seismicity, we indicate by $p(m|m_{0})$ the probability that an event with magnitude $m_{0}$ triggers an earthquake with magnitude *m* inside a temporal window *T*. The epicentral distribution is approximatively given by $\rho \left(\Delta r,m_{0}\right)≃p\left(m|m_{0}\right)G\left(\Delta r,m_{0}\right)$. To evaluate the aftershock epicentral distribution $\rho_{a}\left(\Delta r,m_{M}\right)$ we must restrict to triggered events with $m<m_{0}$ and integrate over all values of $m_{0}\in \left[m_{M},m_{M}+dM\right)$,

(8) $$\rho_{a}\left(\Delta r,m_{M}\right)≃\frac{\int_{m_{M}}^{m_{M}+dM}dm_{0}\int_{m_{th}}^{m_{0}}dmG\left(\Delta r,m_{0}\right)p\left(m|m_{0}\right)}{\int_{m_{M}}^{m_{M}+dM}dm_{0}\int_{m_{th}}^{m_{0}}dmp\left(m|m_{0}\right)}.$$

Conversely, to evaluate $\rho_{f}\left(\Delta r,m_{M}\right)$ we must consider triggered earthquakes with $m>m_{0}$ and since *m* is identified as a mainshock, its magnitude is constrained in the interval $m\in [m_{M},m_{M}+dM)$. Taking into account that the magnitude of the triggering earthquake $m_{0}$, identified as a foreshock, is inside the interval $m_{0}\in \left[m_{th},m\right)$ we obtain

(9) $$\rho_{f}\left(\Delta r,m_{M}\right)≃\frac{\int_{m_{M}}^{m_{M}+dM}dm\int_{m_{th}}^{m}dm_{0}G\left(\Delta r,m_{0}\right)p\left(m|m_{0}\right)}{\int_{m_{M}}^{m_{M}+dM}dm\int_{m_{th}}^{m}dm_{0}p\left(m|m_{0}\right)}.$$

We wish to stress the fundamental difference between Equations (8) and (9) due to the inversion between *m* and $m_{0}$ of the integration range. In Equation (8) the spatial distance is controlled by the kernel $G(\Delta r,m_{0})$ which depends on $m_{0}\in \left[m_{M},m_{M}+dM\right)$ and if dM→0 $\rho_{a}\left(\Delta r,m_{M}\right)≃G\left(\Delta r,m_{M}\right)$. Conversely, in Equation (9) $m_{0}\in \left[m_{th},m_{M}\right)$ and if dM→0, since $p(m|m_{0})$ is an exponential decreasing function of *m*, the integral in Equation (9) is mainly controlled by the contribution from $m≃m_{th}$ which leads to $\rho_{f}\left(\Delta r,m_{M}\right)≃G\left(\Delta r,m_{th}\right)$. As a consequence, in the ETAS model, we expect that $\rho_{f}\left(\Delta r,m_{M}\right)$ only weakly depends on $m_{M}$ differently from $\rho_{a}$. The comparison between Equations (8) and (9) therefore shows that independently of the value of the model parameters, the condition $\rho_{a}\left(\Delta r,m_{M}\right)≃\rho_{f}\left(\Delta r,m_{M}\right)$ cannot be reproduced by the ETAS model. This is confirmed by Figure 4 where the symmetrical behavior $\zeta_{a}\left(\Delta r,m_{M}\right)≃\zeta_{f}\left(\Delta r,m_{M}\right)$, observed in the instrumental catalogs, is not recovered in the ETAS catalogs. Equations (8) and (9) also indicate that $\rho_{a}\left(\Delta r,m_{M}\right)$ is substantially independent of the value of $m_{th}$ whereas $\rho_{f}\left(\Delta r,m_{M}\right)$ is expected to significantly depend on it, in agreement with numerical simulations of the ETAS model [29]. In instrumental catalogs, conversely, the symmetry $\rho_{a}\left(\Delta r,m_{M}\right)≃\rho_{f}\left(\Delta r,m_{M}\right)$ is observed quite independently of the value of $m_{th}$ [29]. This is another important difference of the spatial distribution of foreshocks between instrumental and ETAS catalogs.

### 4.3. Results in the ETASI2 Catalog

Results of the previous section show that ETAS catalogs contain fewer aftershocks than instrumental catalogs. A possible explanation of the foreshock deficit is related to the difficulty to identify small events which occur soon after larger ones as discussed in Section 2.2. This makes instrumental catalog incomplete in the first part of the aftershock sequence. As a consequence, the value of *A*, implemented in the ETAS model to recover the instrumental ratio $n_{aft}\left(m_{M}\right)/n_{main}\left(m_{M}\right)$, is underestimated as well as the ratio of $n_{fore}\left(m_{M}\right)/n_{main}\left(m_{M}\right)$. In this section we take explicitly into account the role of incompleteness by studying aftershocks and foreshocks statistical feature in the ETASI2 catalog.

We have performed extended numerical simulations of the ETASI2 model exploring a wide range of model parameters and evaluated $n_{aft}\left(m_{M}\right)$ and $n_{fore}\left(m_{M}\right)$. Restricting to parameters with $n_{aft}\left(m_{M}\right)/n_{main}\left(m_{M}\right)$ in agreement with the instrumental RSCEC catalog, we find (Figure 3) that, as expected, the incompleteness increases the value of $n_{fore}\left(m_{M}\right)/n_{aft}\left(m_{M}\right)$ which, however, still remains systematically smaller than the value found in instrumental catalogs. For fixed ψ and Δm in Equation (3), the ratio $n_{fore}\left(m_{M}\right)/n_{aft}\left(m_{M}\right)$ does not strongly depend on different choices of σ, as well as on different values of A, p, c and it is always significant smaller than the instrumental one. We also find that $n_{fore}\left(m_{M}\right)/n_{aft}\left(m_{M}\right)$ slightly increases for decreasing Δm and becomes approximately Δm independent for Δm≲0. However, also in this case the value $n_{fore}\left(m_{M}\right)/n_{aft}\left(m_{M}\right)$ is significantly smaller than the one measured in the instrumental catalogs. The origin of this discrepancy is that incompleteness also affects the foreshock number. Indeed, considering a mainshock of magnitude $m_{2}$ anticipated by an event (a foreshock) with magnitude $m_{1}<m_{2}$, incompleteness does not only affect the identification of both $m_{1}$ and $m_{2}$ but it can hide foreshocks with magnitude $m<m_{1}$ occurring between them. Therefore, if the parameters are tuned in order to produce a higher aftershock incompleteness this also reduces the foreshock number and the experimental result is never recovered. In Figure 1 we plot for each instrumental catalog the results of the ETASI2 model for parameters leading to the best agreement for $n_{aft}\left(m_{M}\right)/n_{main}\left(m_{M}\right)$ and minimizing the discrepancy for $n_{fore}\left(m_{M}\right)/n_{main}\left(m_{M}\right)$. Results are obtained assuming ϕ=0.75 and Δm=0.8 for all catalogs, and keeping σ=0.3. Furthermore we use p=1.2 and c=0.01 s. The values of *A* and α producing the best agreement are listed in the caption of Figure 1.

In numerical simulations (not shown) of the ETASI model we find that the spatial distribution of aftershocks in the ETASI2 and in the ETAS model are practically indistinguishable. Indeed, incompleteness typically affects the number of aftershocks but not their spatial distributions. The same observation also holds for the spatial distribution of foreshocks. Therefore, as in the ETAS model, the same deviations from the instrumental finding $\zeta_{a}\left(\Delta r,m_{M}\right)≃\zeta_{f}\left(\Delta r,m_{M}\right)$ is also observed in ETASI2 catalogs.

### 4.4. Results in ETAFS Catalogs

Previous section have shown a systematic deficit of foreshocks in ETAS catalogs which cannot be attributed to the incompleteness of the instrumental catalogs. This motivates the addition of events, different from the ones already implemented in the ETAS catalogs, as in the ETAFS model (Section 2.3). In numerical simulations of the ETAFS model we fix c=100 s and chose the parameters $B,c^{\prime },\alpha^{\prime }$, for each instrumental catalog, in order to reproduce the value $n_{fore}\left(m_{M}\right)/n_{main}\left(m_{M}\right)$, for different $m_{M}$ (see Table 1). We also take explicitly into account aftershock incompleteness, implemented as in the ETASI2 model, and finally generate synthetic catalogs with the same value of $n_{aft}\left(m_{M}\right)/n_{main}\left(m_{M}\right)$ and $n_{fore}\left(m_{M}\right)/n_{main}\left(m_{M}\right)$ as instrumental ones. Figure 3 shows that for all values of $m_{M}$ ETAFS catalogs contains the same number of both aftershocks and foreshocks of instrumental data sets.

Interestingly, adding the “extra” foreshocks allows us to generate numerical catalogs which reproduce also the result $\zeta_{a}\left(\Delta r,m_{M}\right)≃\zeta_{f}\left(\Delta r,m_{M}\right)$, observed in instrumental catalogs (Figure 2). Indeed, in (Figure 4) we compare the average fore-mainshock distance $\zeta_{f}\left(\Delta r,m_{M}\right)$ between the ETAFS and the RSCEC catalog and obtain satisfactory agreement for all values of $m_{M}$.

## 5. Conclusions

We have shown that in four different instrumental regional catalogs, the configuration where a smaller earthquake precedes the occurrence of a larger one occurs more frequently than what expected according to the ETAS model. Furthermore, the average spatial distance between the two earthquakes in instrumental catalogs is significantly larger than predicted by the ETAS model. These results support the idea that the preparatory phase of an earthquake can be accompanied by the nucleation of small earthquakes, a mechanism expected according to the nucleation theory [13,14,15] but not present in the ETAS model. We have therefore presented a novel model which, together with the usual aftershock triggering, assumes that an earthquake can be anticipated by the occurrence of smaller ones. The occurrence probability of these small events is formalized on the basis of statistical empirical features of foreshocks without any a priori physical explanation. The model is supported by its efficiency in reproducing statistical features of both aftershocks and foreshocks in instrumental catalogs.

All properties investigated in this study are obtained by means of a stacking procedure. An interesting point is the behavior expected according to the ETAFS model for the seismic activity before a single large shock. The best-fit parameters of the ETAFS model (Table 1) indicate a small coefficient $\alpha_{f}≃0.5$ in the foreshock productivity law and accordingly the number of foreshock remains relatively small also before large $m_{M}$. For instance, we expect on average less than 8 (m>2) foreshocks the day before a magnitude m=7 mainshock, within a radius of 10 km. This small number implies that for a single mainshock, foreshock activity can at most appear in the form of isolated bursts not leading to an evident systematic increase of the seismic rate. This is consistent with experimental observations, where the inverse Omori-law is obtained only after a stacking procedure and rarely observed inside isolated sequences [59,60].

The agreement with experimental data suggests that the ETAFS model can contribute to a significant improvement of pre-seismic forecasting. A rigorous validation of this point, however, needs to be tested in prospective tests. Unfortunately, a main limitation of the model is that it is not immediately suitable to be implemented in this kind of analysis. Indeed, in order to forecast the occurrence of an earthquake, according to the ETAFS model it is necessary to distinguish foreshock from normal earthquake triggering. An attempt in this direction can be found in [17] where the ETAS occurrence probability is multiplied by an ad-hoc function which gives different weights to aftershock and foreshock clustering. This produces significant gain in the retrospective forecasting of m>6 earthquakes. The nature of foreshocks implemented in the ETAFS model is consistent with this approach promoting further studies on the relevance of foreshocks in seismic forecasting.

## Figures and Tables

**Table 1:** Best parameters of the ETAFS model.

Catalog	A	α	B	$\alpha_{f}$	μ s^−1^
RSCEC	0.084	0.9	0.050	0.54	5.84 × 10^−4^
RNCEC	0.082	0.88	0.033	0.59	4.98 × 10^−4^
ItEC	0.086	0.88	0.052	0.60	5.21 × 10^−4^
JapEC	0.234	0.6	0.160	0.36	5.92 × 10^−3^
